# Measurement Invariance in the Center for Epidemiologic Studies-Depression (CES-D) Scale among English-Speaking Whites and Asians

**DOI:** 10.3390/ijerph18105298

**Published:** 2021-05-16

**Authors:** Tsukasa Kato

**Affiliations:** Department of Social Psychology, Toyo University, 5-28-20 Hakusan, Bunkyo-ku, Tokyo 112-8606, Japan; mtsukasa@hotmail.com; Tel.: +81-3-3945-4542; Fax: +81-3-3945-7626

**Keywords:** Asia, Center for Epidemiologic Studies Depression Scale (CES-D), depressive symptoms, cross-cultural difference

## Abstract

The Center for Epidemiologic Studies Depression (CES-D) Scale has been widely used to measure depressive symptoms. This study compared the measurement invariances for one-, two-, three-, and four-factor models of the CES-D across English-speaking Whites and Asians: White Americans, White Australians, Indians, Filipinos, and Singaporeans. White Americans and Australians, Indians, Filipinos, and Singaporeans English speakers (782 men and 824 women) whose ages ranged from 20 to 79 years, completed the CES-D. They were recruited from the data pool of the 2013 and 2014 Coping and Health Survey. Confirmatory factor analyses indicated that the original four-factor model showed the best fit, compared to the other models. Mean and covariance structure analyses showed that the factor means of the CES-D subscales among Whites were significantly lower than were those among Asians; the score gap was particularly high between Whites and Indians. Additionally, Indians scored the highest on all subscales of the CES-D compared to all other countries. Overall, CES-D scores among Whites were lower than those among Asians.

## 1. Introduction

According to a face-to face household survey of community adults [1], the 12-month prevalence of mood disorders in Asia and America ranges from 1.7% to 3.1% and 4.8% to 9.6%, respectively; depression is a leading cause of disability worldwide. The Center for Epidemiologic Studies Depression (CES-D) Scale [2] is a 20-item self-reported questionnaire designed to measure depressive symptoms among general populations. It has been widely used in many countries with many racial/ethnic groups [3]; indeed, in a list of the 100 most-cited papers of all time by *Nature* in 2014 [4], the article [2] reporting on the development of the CES-D was 51st (*N* = 17,055 citations).

Researchers must address measurement invariance when examining cross-cultural differences in CES-D scores because, without measurement equivalence across ethnic or cultural groups, it is difficult to interpret the differences in observed mean scores meaningfully [5]. Radloff [2] originally proposed a four-factor model for the CES-D, comprising depressed affect, somatic complaints, interpersonal problems, and positive affect. This four-factor structure has been extensively replicated, particularly among Whites [3,6], including Australians [7,8,9]. For example, the National Longitudinal Study of Adolescent Health in the U.S. showed that the original four-factor structure was a better fit compared to the one- and three-factor models [10]. Moreover, the original four-factor structure has been identified in other racial/ethnic groups [11,12], including Asians [13,14,15]. A study [13] using a sample of 1200 for each of five Asian countries—Indonesia, Korea, Myanmar, Sri Lanka, and Thailand—showed that the original four-factor structure was replicated for all countries.

However, many studies have revealed other factor structures for the CES-D in multiple cultural groups, specifically in Asian populations [16,17,18,19,20]. For example, a meta-analysis replicated [3] across African Americans, American Indians, Asians, Whites, and Hispanics, using confirmatory factor analysis (CFA), demonstrated that the original four-factor structure was acceptable across all ethnic groups except for the Asian group (*N* = 65,554, *k* = 16). Two- and three-factor structures have also been cited as other possible factor structures for the CES-D; the two-factor structure comprises negative affect (depressed affect, somatic complaints, and interpersonal problems) and positive affect, whereas the three-factor structure comprises depressed affect and somatic complaints, interpersonal problems, and positive affect.

In addition to measurement invariance, researchers must address complications regarding translation equivalence when examining cross-cultural differences [21]. Indeed, CES-D scores and factor structures were found to be strongly influenced by participants’ language [22]. Therefore, to facilitate accurate comparison, we selected India, the Philippines, and Singapore as English-speaking Asian countries.

People in Asian cultures reported higher scores on the CES-D than those in Western cultures [13,14], particularly for somatic complaints, positive affect, and interpersonal problems. Although somatic symptoms are a common feature of depression in many countries [23], non-Westerners, particularly Asians, tend to show stronger somatic symptoms than Westerners [24,25]. Several explanations for this tendency among Asians have been proposed, one of which concerns how cultural differences in symptom presentation are derived from variations in processing or expressing affect [25]. For example, although Asians tend to pay more attention to somatic symptoms than Westerners, their somatization tends to exhibit lower levels of interoceptive accuracy [25], which is one’s ability to accurately conjecture the magnitude of his or her bodily changes. Regarding positive affect, individuals in Asian cultures tend to avoid or inhibit expressing positive emotions because they are less likely to desire maximization of such emotions and minimization of negative emotions. Instead, they tend to promote a more balanced perspective on positive emotions, in contrast to Westerners, who tend to consider positive emotions to be functional and desirable [24]. Concerning interpersonal problems, research on cross-cultural differences in interpersonal relationships has shown that Asians tend to emphasize respecting and living in harmony with others (collectivism), compared to Westerners [26]. Shared emotions in collectivists, including Asians, involve ensuring that others share the concern and behave accordingly, whereas individualist cultures, including Americans and Australian, involve sharing of information. As such, Asians are more likely than Westerners to regard interpersonal problems as important and tend to report more interpersonal problems. Lastly, concerning depressed affect, some researchers [27] suggest that Asians tend to exhibit somatic symptoms rather than psychological symptoms, including depressed affect. However, the emphasis on somatic symptoms is not a minimization or denial of depressed affect, and that cultural differences in depressed affect between Westerners and non-Westerners are attributable to variations in constituent symptoms rather than the actual level of depressed affect [28]. Specifically, Chinese psychiatric outpatients reported less hopelessness among psychological symptoms according to a clinical interview than Euro-Canadian psychiatric outpatients but reported greater suppressed emotions and depressed mood [28]. Indeed, Asians report higher levels of depressed affect than do Westerners [13,14].

The present study compared the measurement invariances for one-, two-, three-, and four-factor models and scores of the CES-D between Asians and Westerns. Few studies have examined cross-cultural differences in CES-D scores between Whites and Indians, Filipinos, or Singaporeans, particularly using the same language. Nevertheless, given these previous theoretical studies on cross-cultural differences in self-reported depressive symptoms, we hypothesized that Whites would report lower CES-D scores than Asian groups.

## 2. Methods

### 2.1. Participants and Procedures

Two surveys, the 2013 and 2014 Coping and Health Survey [29,30,31] were conducted using web-based panels of the polling organization, Rakuten Research. They comprised more than 40.12, 8.26, 9.44, 1.60, and 3.92 hundred-thousand members in the U.S., Australia, India, the Philippines, and Singapore, respectively, who had registered and received one ID per person. Participants in the U.S. and Australia and those in India, Philippines, and Singaporean were selected from the data pools of the 2013 [29,30] and 2014 Coping and Health Survey [31], respectively. The data pools of the former and latter projects were 500 Americans Australians each, and 300 Indians, Filipinos, and Singaporeans each, respectively. The details of the survey were sent to potential participants, who were English speakers and ranged in age from 20 to 79 years, through an e-mail. The data in these surveys were collected so that the sample was almost evenly divided by gender and age in each country in each survey.

Participants (782 men and 824 women) in the present study included White Americans and Australians, Indians, Filipinos, and Singaporeans. The demographic characteristics of each sample are shown in Table 1. Although Singapore has three main ethnic groups―Chinese, Malay, and Indian―only Chinese and Malay were selected for this study to avoid overlap with the Indian sample. There were three Indiana participants.

### 2.2. Measures

In addition to the American population [2], the reliability and validity of CES-D scores have been established in Australian [7,8,9], Indian [17], Filipino [18,19,32], and Singaporean [33,34] populations. Additionally, the reliability and validity of CES-D scores obtained through Internet surveys have been supported [33,35,36]. Participants in this study rated each of the 20 CES-D items according to their experiences within the past week on a 4-point Likert scale ranging from 0 (rarely or none of the time, less than 1 day) to 3 (most or all the time, 5–7 days).

### 2.3. Data Analyses

First, we performed separate CFAs using the maximum likelihood method to test the fitness of the one-, two-, three-, and four-factor models. Second, the measurement invariances of the four models across the five samples were tested using CFA, which comprises the assessment of configural invariance—equivalence of factor structure across groups—metric invariance—equivalence of factor loadings across groups—and scalar invariance—equivalence of intercepts across groups. The configural invariance served as a baseline model. The standardized root-mean-square residual (SRMR; the most sensitive to mis-specified factor covariances), the comparative fit index (CFI; the most sensitive to mis-specified factor loadings), and the root-mean-square error of approximation (RMSEA; a measure of lack of fit per degree of freedom) were used as fit indices. According to the criterion proposed by Tanaka [37], CFI values of 0.95 or greater are considered good fits, whereas CFI values greater than 0.90 are acceptable. RMSEA values of 0.06 or lower are optimal while a SRMR value of 0.08 or lower is acceptable. The expected cross-validation index is used to compare the fit of different models; the model with the smallest positive values is preferred. As chi-square statistics are known to be sensitive to sample size, the ratio of changes in chi-square and degrees of freedom (Δχ^2^/Δ*df*) and changes in CFI (ΔCFI) and SRMR (ΔSRMR) were used to compare the models. Models with a ΔCFI of 0.01—supplemented by 0.030 or lower in ΔSRMR or 0.015 or lower in ΔRMSEA [38]—and a Δχ^2^/Δ*df* of less than or equal to five [39] would have a good fit to the data when CFA was conducted with a sample size of 1606. Additionally, cross-cultural differences in CES-D scores were compared using factor means between countries. Data were analyzed using SPSS version 22 (IBM, Armonk, NY, USA) and AMOS 22.0 (IBM, Armonk, NY, USA).

## 3. Results

The mean item and total scores of the CES-D by sample are shown in Table 2. Total CES-D scores were ranked in descending order as follows: Indians, Singaporeans, Filipinos, and White Americans and Australians. The fit indices in CFAs of all models by sample are shown in Table 3. In White American and Australian samples, all other models excluding the one-factor model satisfied the acceptable cutoff criteria (CFI ≥ 0.90 with SRMR ≤ 0.080 and RMSEA ≤ 0.080). Among Indians, Filipino, and Singaporeans, only the four-factor model met the acceptable cutoff criteria. The one-factor model showed a poor fit to the data for all samples. The results on the CFAs indicated that only the four-factor model was acceptable across all samples.

CFAs were used to test the measurement invariances of the four-factor model across all samples (Table 4). The results of all models excluding the four-factor model are shown in S1 and S2 (Supplementary materials). The respective fit values in metric invariance were acceptable in the four-factor model based on the cutoff criteria (Δχ^2^/Δdf ≤ 5.0 and ΔCFI ≤ 0.01 with ΔSRMR ≤ 0.030 or ΔRMSEA ≤ 0.015). However, ΔCFIs in scalar invariance were unsatisfied; although, the other fit values in scalar invariance were satisfied in the four-factor model. Therefore, to establish partial scalar invariance in the four-factor model, the constraints on loadings and intercepts of items 4, 7, 10, 11, 16, 17, 18, 19, and 20 were released based on Steenkamp and Baumgartner’s [40] recommendation—ideally, more than half items on a factor should be invariant. The results showed that fit indices for the partial scalar invariance in the four-factor model were adequate.

Finally, factor means, which were corrected for noninvariant items, in the four-factor model were compared between samples. Factor means for somatic complaints showed that White Australians (*M* = 0.35 and SE = 0.06, *z* = 5.53, *p* < 0.001) and Americans (*M* = 0.40 and SE = 0.06, *z* = 6.38, *p* < 0.001) reported lower scores compared to Indians. Regarding depressed affect, the factor mean among White Australians was lower as compared to that among Singaporeans (*M* = 0.16 and SE = 0.06, *z* = 2.97, *p* = 0.003), Filipinos (*M* = 0.22 and SE = 0.05, *z* = 4.10, *p* < 0.001), and Indians (*M* = 0.37 and SE = 0.06, *z* = 6.12, *p* < 0.001), and the factor mean among White Americans was lower than that for Indians (*M* = 0.24 and SE = 0.06, *z* = 3.65, *p* < 0.001). Factor means for (low) positive affect showed that White Australians (*M* = 0.21 and SE = 0.05, *z* = 4.12, *p* < 0.001) and Americans (*M* = 0.20 and SE = 0.05, *z* = 3.94, *p* < 0.001) reported lower scores than Singaporeans. Regarding interpersonal problems, the factor mean among White Australians was lower than that among Singaporeans (*M* = 0.20 and SE = 0.05, *z* = 4.06, *p* < 0.001), Filipinos (*M* = 0.20 and SE = 0.05, *z* = 3.96, *p* < 0.001), and Indians (*M* = 0.37 and SE = 0.06, *z* = 6.24, *p* < 0.001), and the factor mean of White Americans was lower than that of Indians (*M* = 0.25 and SE = 0.06, *z* = 4.06, *p* < 0.001).

## 4. Discussion

The four-factor CES-D has been well validated [5,6,11,12], which has also been replicated by our findings. Previous studies validating the four-factor structure of the CES-D in Asian countries largely used Chinese samples [14,15], with a non-English-language version of the CES-D. However, this study used Indian, Filipino, and Singaporean samples with an English-language version of the CES-D. Therefore, our findings that the four-factor structure of the CES-D was established in Asian populations may contribute to future research on the CES-D with Asians. However, our findings cannot exclude the possibility of other-factor structures of the CES-D, which have been proposed in Asian cultures [16,17,18,19,20].

Although our findings replicated the four-factor structure of the CES-D, we did not examine its clinical validity or usefulness. In Asian cultures, positive items in self-report questionnaires on depression may not be useful as markers of depression [24]. For example, European and Asian Americans born in the U.S. reported that the intensity of positive emotions was negatively associated with depressive symptoms; however, this was not the case with Asians who had immigrated into the U.S. [41]. Moreover, for Asians, some studies [15] about the CES-D have also questioned the clinical usefulness of the positive affect subscale. For example, in a study of Chinese patients [15], excluding the positive affect items provided a better screening tool for depression as compared to the original CES-D.

Before we compared CES-D scores among the samples, CFAs conducted to test the measurement invariance across the five samples, revealed that the goodness-of-fit statistics of the four-factor model were acceptable. This result indicated that the mean CES-D subscale scores of the four-factor model were comparable across the five samples. We hypothesized that CES-D scores among Whites would be higher than those among Asians. Total CES-D scores were ranked in descending order as follows: Indians, Singaporeans, Filipinos, White Americans and Australians. Factor means for somatic complaints were lower among White Americans and White Australians than those among Indians. Furthermore, factor means for depressed affect were lower among White Americans than those among Indians, whereas White Australians showed lower scores than all Asian samples. Factor means for low positive affect among White Americans and Australians were significantly lower than were those among Singaporeans. Factor means for interpersonal problems were lower among White Americans than among Indians, whereas White Australians showed lower scores than all the Asian samples. In sum, overall, the CES-D subscales scores among Whites were lower than those among Asians, with the gap being greater for Whites and Indians. Additionally, overall, factor means of all subscales among Indians were highest among all Asians; although, we did not hypothesize about differences in CES-D scores in this study. These findings are addressed in the following paragraphs.

First, this study showed that overall, Indians reported the highest CES-D scores when compared to other samples, particularly concerning somatic complaints. Somatic symptoms are core diagnostic symptoms of depression for Indians and are the presenting complaints for 97% of Indian primary care patients [42]. One possible reason for this is that yoga plays a central and definitive role in many Indians’ lives. Yoga is a spiritual and physical discipline in Hinduism, which is the main religion of India; although a more spiritual meditation than physical exercise, it helps Indians focus on bodily changes in the moment. Therefore, Indian people may have greater somatic awareness and show stronger somatic symptoms than people in other countries.

Second, although India, the Philippines, and Singapore had been colonized by countries in Europe and America, the colonial periods in the Philippines and Singapore were longer than India; thus, Filipino and Singaporean cultures have been more heavily influenced by Western culture than the Indian culture. Such cultural differences might influence the differences in CES-D scores in our findings. Some studies found no significant differences between Filipinos and Whites concerning CES-D scores; although, to our knowledge, no study had examined differences in CES-D scores between them. A previous study [32] conducted on college students in Hawaii showed no significant differences in CES-D total scores between European Americans and Filipinos; although, Filipinos’ scores (*M* = 16.51, SD = 10.96) were somewhat higher than were European Americans’ scores (*M* = 15.09, SD = 9.70). Additionally, a previous study [34] suggested that CES-D total score among Indians was higher than that among Singaporeans; although, to our knowledge, the current study was the first to examine differences in CES-D scores between Filipino, Singaporean, and Indian populations.

### Limitations

Some limitations must be considered when interpreting our findings. First, the generalization or representativeness of the current findings to other samples may be low. Of many Asian countries, we selected only three to address the translation equivalence of the CES-D. Furthermore, we did not obtain data from White Europeans or Canadians. In other words, our samples are not entirely representative of Asian or White samples. Additionally, the Asian samples in this study may not have been representative of Indians, Filipinos, or Singaporeans, as we used only English-speaking participants. Moreover, the CES-D scores were obtained through a web-based survey; the data may differ if collected via other methods, such as by telephone, interviews, or paper-based self-report. A previous study [35] conducted in the Netherlands found that the mean CES-D total score among adolescents recruited through the Internet (*M* = 15.4, SD = 11.8) was significantly higher than that of adolescents recruited through schools (*M* = 9.7, SD = 8.4). Further, scores of depressive symptoms obtained through the Internet were greater than those obtained by paper-based self-report [43].

Second, although the results in this study suggested that the four-factor was an acceptable structure for the CES-D, this does not suggest that the four-factor structure is more useful for clinical or diagnostic research on depression than are the other-factor structures. Additionally, in other ethnic groups, the one-, two-, three-, or other-factor models may be a better structure for the CES-D.

Third, the findings on the differences in CES-D scores need to be interpreted with caution. Partial scalar invariance in the four-factor model was acceptable in our samples, but not scalar invariance. Standards for partial invariance are inconsistent [44]; however, partial invariance has been reported for approximately one-third of tests [44]. The findings on the differences in CES-D scores contribute to research on the CES-D.

Finally, although our results showed that Asians reported higher CES-D scores than Whites, the prevalence of depression in Asians is not necessarily higher than that among Westerners when diagnosed via an interview with a psychiatrist. The World Health Organization [45] reported the prevalence of current depression, according to Composite International Diagnostic Interviews, was 9.1% in India, 4.0% in China, 6.3% in the U.S., and 4.7–16.9% among Western Europeans. This contradiction may suggest that cross-cultural differences in CES-D scores do not necessarily correspond to those in the prevalence of depression as diagnosed by a psychiatrist.

## 5. Conclusions

The CES-D has been widely and frequently used to measure depressive symptoms in the general population. Although an appropriate structure of the CES-D was different in different cultures, the four-factor model was most adequate across all cultures. Despite some limitations, our findings among English-speaking Whites and Asians indicate that the mean CES-D scores in Whites were lower than those in Asians; the gap was particularly high among Indians.

### Declarations

All participants were selected from the data pools of the 2013 [29,30] and 2014 Coping and Health Survey [31], as described in the Methods section. The Coping and Health Survey was administered for several purposes. The present study used the CES-D, which was a part of the data obtained by the 2013 and 2014 Coping and Health Surveys. The mean CES-D scores in the U.S., Australian, and Chinese samples were reported by Kato [29] using the data from the 2013 Coping and Health Surveys. The mean CES-D scores in the Indian, Filipino, and Singaporean samples were reported by Kato [31] using the data from the 2014 Coping and Health Survey. Based on PLoS ONE editorial policies regarding the sharing of materials and data, the raw data obtained from the 2013 Coping and Health Survey were made freely available (Supporting Information: Dataset S1).

## Figures and Tables

**Table 1:** Demographic characteristics of each sample.

Characteristic	American	Australian	Indian	Filipino	Singaporean
*N*	393	425	223	286	279
Mean age	47.2	47.4	39.4	36.0	36.1
SD	15.3	14.4	11.1	11.5	10.5
Women (%)	51.1	53.9	50.2	50.3	49.5
Married (%)	54.5	53.9	70.4	46.2	45.5
Never married (%)	26.5	30.6	28.4	50.7	52.3
Divorced/separated/widowed (%)	19.1	15.5	1.3	3.1	2.2
Ethnic group (%)	White (100)	White (100)	Punjabi (17.0) Tamil (14.8) Gujarati (14.3) Bengali (13.5) Marathi (13.5) Telugu (12.6) Kannada (8.5) Malayali (5.8)	Filipino (98.3) Half Filipino (1.7)	Chinese (95.0) Malay (5.0)

**Table 2:** Item scores and alphas of the CES-D and the mean CES-D total scores by sample.

Item		American	Australian	Indian	Filipino	Singaporean
*n*		393	425	223	286	279
Somatic Complaints						
1	Bothered	0.64	0.63	0.98	0.75	0.71
2	Appetite	0.58	0.50	0.91	0.51	0.57
5	Mind	0.81	0.74	1.08	0.94	0.79
7	Effort	0.91	0.84	1.32	1.34	1.20
11	Sleep	1.02	1.10	1.01	0.82	0.86
13	Talk	0.76	0.75	1.23	0.98	0.89
20	Get going	0.75	0.79	0.91	0.64	0.76
	Total	5.47	5.34	7.44	5.98	5.79
	SD	4.52	4.50	4.69	4.32	4.58
	*α*	0.83	0.84	0.81	0.81	0.85
Depressed Affect						
3	Blues	0.78	0.66	1.02	0.76	0.73
6	Depressed	0.83	0.68	1.03	0.85	0.80
9	Failure	0.66	0.48	0.93	0.70	0.78
10	Fearful	0.65	0.43	1.00	0.80	0.75
14	Lonely	0.79	0.64	1.03	0.88	0.87
17	Cry	0.47	0.35	0.84	0.63	0.53
18	Sad	0.78	0.61	0.96	0.92	0.77
	Total	4.97	3.86	6.80	5.53	5.25
	SD	5.28	4.70	5.24	5.02	5.17
	*α*	0.91	0.90	0.88	0.92	0.91
(Low) Positive Affect						
4	Good	1.28	1.30	1.50	1.65	1.68
8	Hopeful	1.41	1.48	1.26	1.12	1.65
12	Happy	1.22	1.18	1.16	1.09	1.47
16	Enjoy	1.22	1.18	1.16	0.95	1.56
	Total	5.13	5.15	5.08	4.82	6.37
	SD	3.60	3.63	3.05	2.86	3.21
	*α*	0.83	0.85	0.70	0.68	0.80
Interpersonal Problems						
15	Unfriendly	0.57	0.37	0.89	0.59	0.66
19	Dislike	0.58	0.49	0.83	0.72	0.67
	Total	1.15	0.86	1.72	1.31	1.33
	SD	1.52	1.29	1.73	1.59	1.53
	*α*	0.71	0.63	0.72	0.76	0.77
CES-D Total Score						
	Mean	16.72	15.20	21.04	17.65	18.73
	SD	12.55	11.72	11.08	10.78	11.40
	*α*	0.93	0.92	0.88	0.90	0.91

**Table 3:** Confirmatory factor analysis for the five models of the CES-D by sample.

Model	*χ* ^2^	*df*	ECVI	90% CI	CFI	SRMR	RMSEA
White American (*n* = 394)							
One-factor	894.04	170	2.59	2.36–2.84	0.83	0.079	0.104
Two-factor	497.76	169	1.48	1.32–1.66	0.92	0.051	0.070
Three-factor	474.02	167	1.43	1.27–1.60	0.93	0.049	0.068
Four-factor	414.15	164	1.39	1.25–1.56	0.94	0.043	0.062
White Australian (*n* = 425)							
One-factor	1031.89	170	2.72	2.49–2.96	0.81	0.082	0.109
Two-factor	485.44	169	1.34	1.19–1.50	0.93	0.045	0.066
Three-factor	470.88	167	1.31	1.17–1.48	0.93	0.044	0.066
Four-factor	416.79	164	1.29	1.16–1.45	0.94	0.042	0.060
Indian (*n* = 223)							
One-factor	571.29	170	3.11	2.80–3.46	0.80	0.092	0.103
Two-factor	394.81	169	2.15	1.90–2.43	0.89	0.079	0.078
Three-factor	393.34	167	2.16	1.92–2.44	0.89	0.079	0.078
Four-factor	379.63	164	2.31	2.07–2.58	0.90	0.077	0.077
Filipino (*n* = 286)							
One-factor	701.00	170	2.88	2.61–3.18	0.81	0.083	0.105
Two-factor	505.69	169	2.20	1.98–2.45	0.88	0.080	0.084
Three-factor	483.59	167	2.14	1.92–2.38	0.89	0.080	0.082
Four-factor	456.62	164	2.07	1.86–2.30	0.90	0.078	0.079
Singaporean (*n* = 279)							
One-factor	833.55	170	3.43	3.12–3.77	0.79	0.102	0.118
Two-factor	498.01	169	2.23	2.00–2.49	0.90	0.071	0.084
Three-factor	475.27	167	2.16	1.94–2.41	0.90	0.069	0.080
Four-factor	465.90	164	2.15	1.93–2.40	0.91	0.068	0.080

Note. ECVI, CFI, SRMR, and RMSEA are expected cross-validation index, comparative fit index standardized root-mean-square residual, and root-mean-square error of approximation, respectively.

**Table 4:** Mean and covariance structure analysis for the four-factor model.

Model	*χ* ^2^	*df*	CFI	SRMR	RMSEA	Δ*χ*^2^	Δ*df*	Δ*χ*^2^/Δ*df*	ΔCFI	ΔSRMR	ΔRMSEA
Configural invariance	2133.45	820	0.920	0.039	0.032						
Metric invariance	2350.56	884	0.911	0.046	0.032	217.10	64	3.39	0.009	0.007	0.001
Scalar invariance	2851.04	964	0.885	0.046	0.035	717.59	144	4.98	0.035	0.007	0.003
Partial scalar invariance	2405.99	896	0.910	0.048	0.032	272.54	76	3.59	0.010	0.009	0.001

Note. CFI, SRMR, and RMSEA are comparative fit index, standardized root-mean-square residual, and root-mean-square error of approximation, respectively.

## Data Availability

Not applicable.

## Supplementary Materials

The following are available online at https://www.mdpi.com/article/10.3390/ijerph18105298/s1, Table S1: Mean and covariance structure analysis for other models excluding four-factor model of the CES-D, Table S2: Differences in factor means between samples for the four-factor model.
