# Supplementary material for: Measurement Invariance in the Center for Epidemiologic Studies-Depression (CES-D) Scale among English-Speaking Whites and Asians
Source: Int J Environ Res Public Health. 2021 May 16;18(10):5298. doi: 10.3390/ijerph18105298 (PMC8156289; doi:10.3390/ijerph18105298)
Supplement: Supplementary file 1 [file ijerph-18-05298-s001.zip › ijerph-1154768-supplementary.pdf]

Supplementary materials

Table S1. Mean and covariance structure analysis for other models excluding four-factor model of the CES-D.

**Model**	χ^2	df	**CFI**	**SRMR**	**RMSEA**	$\Delta\chi^2$	Δdf	$\Delta\chi^2/\Delta df$	**ΔCFI**	**ΔSRMR**	**ΔRMSEA**
					One-factor model						
Configural invariance	4031.92	850	0.807	0.079	0.048						
Metric invariance	4479.59	926	0.785	0.108	0.049	447.67	76	5.89	0.022	0.002	0.001
Scalar invariance	5035.86	1006	0.756	0.109	0.050	1003.94	156	6.44	0.051	0.003	0.002
					Two-factor model						
Configural invariance	2382.03	845	0.907	0.051	0.034						
Metric invariance	2630.53	917	0.896	0.057	0.034	248.50	72	3.45	0.011	0.006	0.001
Scalar invariance	3131.50	997	0.871	0.057	0.037	749.47	152	4.93	0.036	0.006	0.003
					Three-factor model						
Configural invariance	2297.43	835	0.911	0.049	0.033						

Metric invariance	2519.95	903	0.902	0.055	0.033	212.52	68	3.13	0.009	0.006	0.001
Scalar invariance	3026.11	983	0.876	0.055	0.036	728.68	148	4.92	0.035	0.006	0.003

Note. *N* = 1,606. CFI, SRMR, and RMSEA are comparative fit index, standardized root-mean-square residual, and root-mean-square error of approximation, respectively. CES-D is the Center for Epidemiologic Studies-Depression.

Table S2. Differences in factor means between samples for the four-factor model.

CES-D subscale	Main finding
Somatic complaints	Australian, Americans, Filipinos, and Singaporeans < Indians
Depressed affect	Australian < Americans, Singaporeans, and Filipinos < Indians
(low) Positive affect	Australian, Americans, Filipinos, and Indians < Singaporeans
Interpersonal problems	Australian < Americans, Singaporeans and Filipinos < Indians

Note. CES-D is the Center for Epidemiologic Studies-Depression.
